# Pediatric spinal ependymoma with chromothripsis of chromosome 6: a case report and review of the literature 

**DOI:** 10.1186/s13256-023-04283-4

**Published:** 2024-02-14

**Authors:** Keela R. Scott, Melissa A. Gener, Elena A. Repnikova

**Affiliations:** 1 Department of Pathology & Anatomical Sciences, University of Missouri—Columbia, 1 Hospital Drive M263, MSB, Columbia, MO 65212 USA; 2 Department of Pathology & Laboratory Medicine Children’s Mercy Kansas City, University of Missouri—Kansas City School of Medicine, Kansas City, MO 64108 USA

**Keywords:** Spinal ependymoma, Ependymoma, Pediatrics, Chromothripsis, Neurofibromatosis type 2

## Abstract

**Background:**

Ependymomas are the third most common central nervous system tumor in the pediatric population; however, spinal ependymomas in children are rare. Ependymomas affecting the spinal cord most frequently occur in adults of 20–40 years of age. The current World Health Organization classification system for ependymomas is now composed of ten different entities based on histopathology, location, and molecular studies, with evidence that the new classification system more accurately predicts clinical outcomes.

**Case presentation:**

We present the case of a 16-year-old Caucasian female patient with a history of type 2 neurofibromatosis with multiple schwannomas, meningioma, and spinal ependymoma. Chromosome analysis of the harvested spinal ependymoma tumor sample revealed a 46,XX,−6,+7,−22,+mar[16]/46,XX[4] karyotype. Subsequent OncoScan microarray analysis of the formalin-fixed paraffin-embedded tumor sample confirmed + 7, −22 and clarified that the marker chromosome represents chromothripsis of the entire chromosome 6 with more than 100 breakpoints. Fluorescent in situ hybridization and microarray analysis showed no evidence of *MYCN* amplification. The final integrated pathology diagnosis was spinal ependymoma (central nervous system World Health Organization grade 2 with no *MYCN* amplification.

**Conclusion:**

This case adds to the existing literature of pediatric patients with spinal ependymomas and expands the cytogenetic findings that may be seen in patients with this tumor type. This case also highlights the value of cytogenetics and microarray analysis in solid tumors to provide a more accurate molecular diagnosis.

## Background

Ependymomas (EPNS) can develop anywhere along the neuroaxis, and recent data suggest they may arise from radial glial cells [1]. They are the third most common central nervous system (CNS) tumor in the pediatric population [2], representing approximately 6–12% of brain tumors in children and 15% of spinal cord tumors across all age groups, with the vast majority occurring in adults [3]. However, pediatric spinal ependymomas, specifically, are rare and there is limited information regarding the true incidence and behavior of these entities. One study based on Surveillance, Epidemiology, and End Results (SEER) data identified 279 pediatric cases of spinal ependymomas from 2004 to 2014. They concluded that invasive tumors conferred a worse prognosis and, interestingly, higher grade tumors were not clearly correlated with decreased overall survival rates [4].

The complexity of ependymomas and how they develop is further acknowledged by the major change in the 2021 World Health Organization (WHO) classification system, with more emphasis being placed on the molecular characteristics of these tumors. The current WHO classification system for ependymomas is now composed of ten different entities based on histopathology, location, and molecular studies, with evidence that the new classification system more accurately predicts clinical outcomes [5, 6].

We present the case of a pediatric patient with a clinical diagnosis of neurofibromatosis type 2 (NF2) and early onset spinal ependymoma with an extensive genetic analysis of the tumor.

## Case presentation

A 16-year-old Caucasian female with a clinical diagnosis of NF2 presented to the neurosurgery clinic for follow-up of previously identified lesions including bilateral vestibular schwannomas, meningioma status postresection, and an enlarging cervicothoracic intramedullary lesion. At the time of initial diagnosis, the patient presented with bilateral vestibular schwannomas in addition to enhancing tumors involving the anterior aspect of the cervical spinal cord, foramen magnum, and the left cavernous sinus. There was no family history of NF2 or other genetic neurological conditions. Of note, the patient’s family declined germline *NF2* testing. The physical exam was significant for postoperative left foot drop following resection of the right frontal meningioma and a right frontal surgical site with well-approximated skin and thick overlying scabbing, but no signs of infection.

**Fig. 1:**
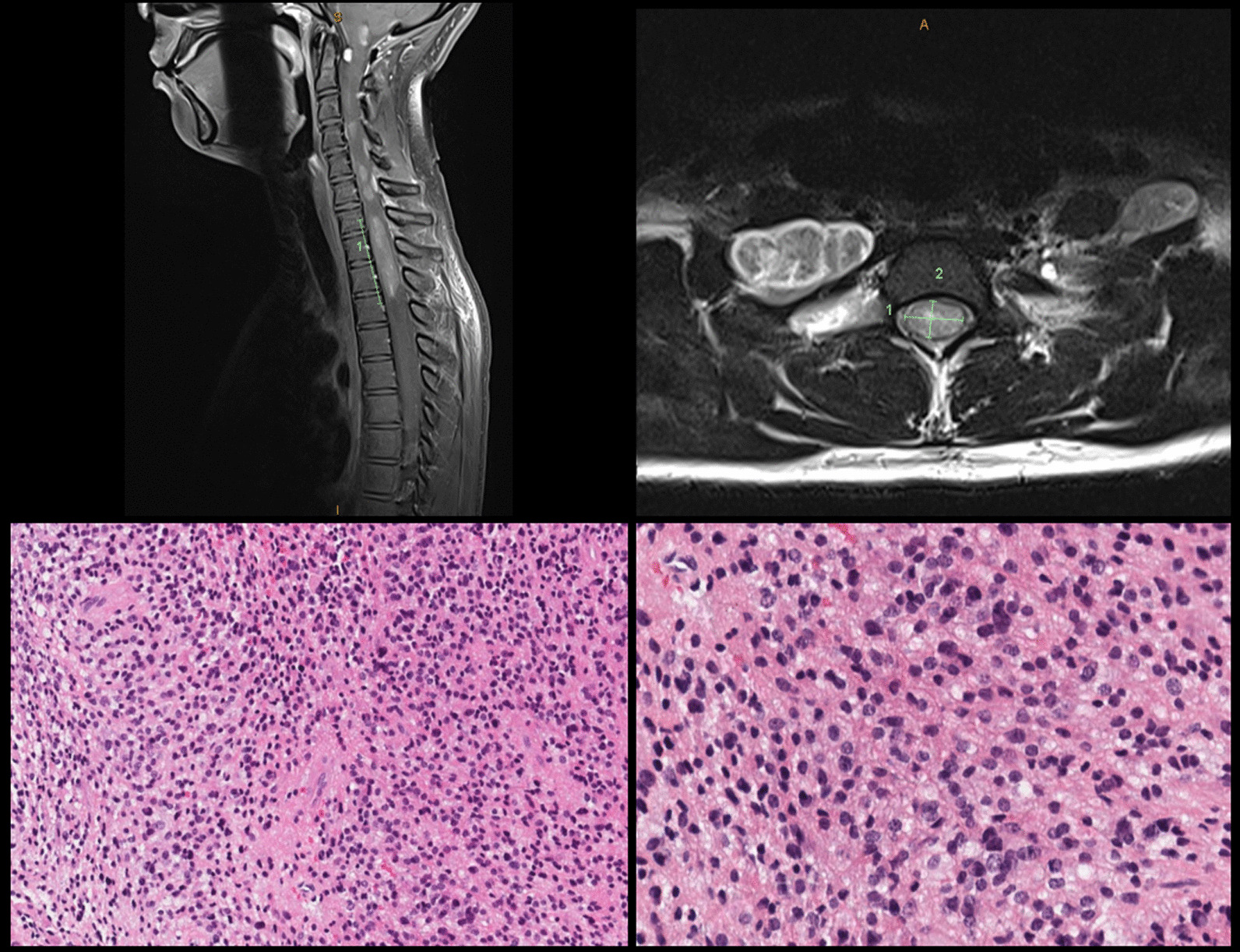
T1-weighted sagittal C-spine MRI: predominantly hypointense 0.9 × 1.4 × 4.8 cm (AP × TRV × CC) intramedullary tumor extending from upper C1 to upper T3 level (top left). T2-weighted transverse C-spine MRI: expansion of the dominant tumor causes the cord to completely fill the spinal canal from C7 through T2 (top right). Characteristic perivascular pseudorosettes [hematoxylin and eosin (H&E) stain ×100 magnification) (bottom left). Isomorphic glial tumor cells with round to oval nuclei in a dense fibrillary glial matrix (H&E ×200 magnification) (bottom right)

Magnetic resonance imaging (MRI) of the cervical spine revealed a 4.8 × 1.4 × 0.9 cm [cranial–caudal (CC) × transverse (TRV) × anterior–posterior (AP)] intramedullary tumor extending from the upper C1 to upper T3 level with a small focus of hydromyelia caudal to the tumor at the T3 level and ill-defined patchy enhancement after contrast (Fig. 1). There was progressive enlargement totaling approximately 2.0 cm (CC) over the course of 10 months when compared with previous imaging studies over that time period. The lesion caused the spinal cord to completely fill the spinal canal from the C7 level through the T2 level. Of note, there were at least three smaller, clustered intramedullary tumors extending from the C4 level through the C6 level with evidence of enlargement as well. Given the progressive enlargement of the dominant intramedullary lesion, the decision was made to undergo debulking of the tumor for pathological diagnosis as well as possible initiation of treatment.

**Fig. 2:**
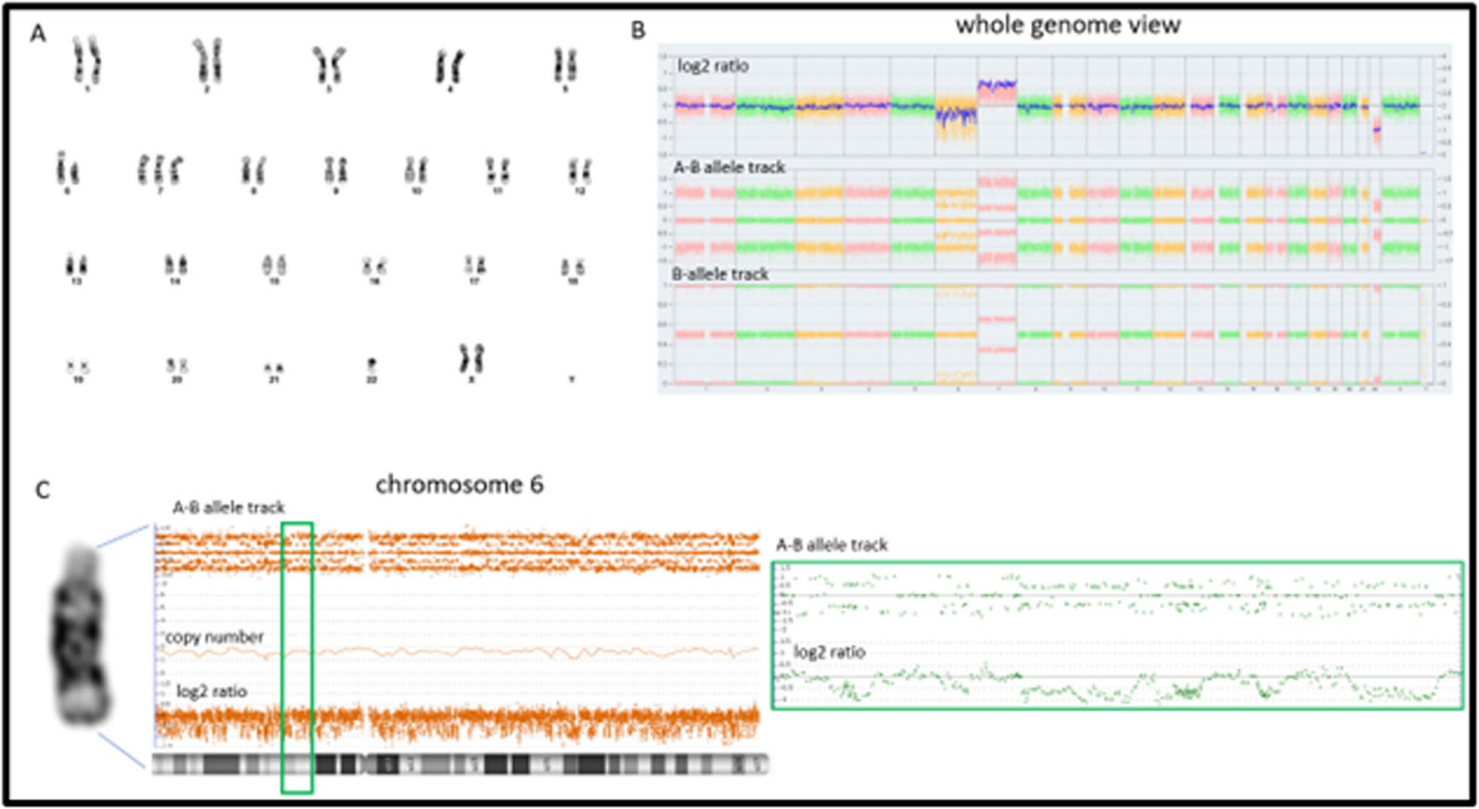
**A** Abnormal karyotype of the harvested spinal ependymoma shows a derivative chromothriptic chromosome 6, extra copy of chromosome 7, and loss of chromosome 22 in 80% of the examined cells. **B** Whole genome ThermoFisher SNP microarray from FFPE tumor confirms gain of chromosome 7, loss of chromosome 22, and numerous regions with copy number losses over the entire chromosome 6. **C** Close up examination of chromosome 6 shows chromothripsis spanning from pter to qter and involving more than 100 breakpoints. A–B allele frequency track appears as 5 tracks with the log_2_ ratio falling slightly below 2. Upon close inspection (green boxed region), it appears that the A–B allele track represents alternating regions, with three and two tracks consistent with normal and deleted regions, respectively

Subsequently, the patient underwent C7–T1–T2–T3 laminectomies for decompression of the spinal cord and resection of the dominant intradural, intramedullary spinal cord tumor. Pathology examination revealed spinal ependymoma, CNS WHO grade 2 (Fig. 1). Immunohistochemistry staining for H3K27me3 showed retained nuclear staining consistent with the normal/wild-type immunophenotype. Fluorescence in situ hybridization (FISH) of the tumor was performed at an outside reference laboratory, which showed no evidence of *MYCN* amplification. Chromosome analysis of the harvested spinal ependymoma tumor sample [7] revealed a 46,XX,−6,+7,−22,+mar[16]/46,XX[4] karyotype. Subsequent OncoScan microarray analysis of the formalin-fixed paraffin-embedded (FFPE) tumor confirmed +7,−22 and clarified that the marker chromosome represents chromothripsis of the entire chromosome 6, involving more than 100 breakpoints, which was visualized as a complex pattern of alternating copy number changes (Fig. 2). Microarray also confirmed no evidence of *MYCN* amplification in agreement with FISH results. The final integrated pathology diagnosis is spinal ependymoma (CNS WHO grade 2) with no *MYCN* amplification.

The patient made a full, uneventful recovery from surgery. She was also seen by the hematology–oncology team with specialization in neuro-oncology. There have been lengthy discussions regarding possible further treatment options including mitogen-activated protein kinase (MEK) inhibitors and mammalian target of rapamycin (mTOR) inhibitors in which the final decision upon which treatment to initiate has yet to be determined.

## Discussion and conclusion

NF2 is a genetic neurocutaneous disorder characterized by the formation of particular lesions throughout both the central and peripheral nervous systems. The syndrome is inherited in an autosomal dominant fashion, although approximately 50% of patients acquire a de novo loss of function variant and approximately 30% of patients are mosaic for the disease-causing loss of function variant in the *NF2* gene [8, 9]. The incidence of NF2 is approximately 1 in 25,000 individuals, and there is considerable variability in presentation and severity [10]. The *NF2* gene is a tumor-suppressor gene localized to chromosome 22q12.1, which produces a protein called merlin. In turn, merlin regulates the downstream signaling of many pathways including, but not limited to, RAS/RAF/MEK/ERK, PI3K/AKT, Rac/PAK/JNK, WNT/β-catenin, mTORC2, SRC/FAK, and others, which are responsible for promoting cellular proliferation and growth [11, 12]. The hallmark feature of NF2 is bilateral vestibular schwannomas, which occur in more than 95% of patients [10]; however, these patients are also susceptible to other tumors such as meningiomas, ependymomas, and schwannomas of other cranial nerves, spinal roots, or peripheral nerves [11, 12].

In children, tumors of the spinal cord are rare, accounting for less than 10% of all pediatric CNS tumors, with ependymomas constituting approximately 30% of those [13]. In patients with NF2, 33–53% of patients develop ependymomas typically in the cervical cord or cervicomedullary junction; however, there is limited literature on the true incidence of spinal ependymomas specifically within the pediatric population [14]. One study based on Surveillance, Epidemiology, and End Results (SEER) data identified 279 pediatric cases of spinal ependymomas from 2004 to 2014 [4]. Another study by Benesch *et al.* [15] identified 29 pediatric patients with spinal ependymomas between 1991 and 2007 from the pediatric German brain tumor studies Hirntumor (HIT) 1991 and HIT 2000, of whom four patients had NF2. At our institution, the case we present is the first case of spinal ependymoma in a pediatric patient in the past 20 years.

The 2021 WHO classification system has redefined the classification of ependymal tumors with the inclusion of molecular phenotypes. Ependymomas of the spinal cord can now be classified into four subtypes: spinal ependymoma (SP-EPN), spinal ependymoma with *MYCN* amplification (SP-MYCN), myxopapillary ependymoma (MPE), and subependymoma (SE). It is noteworthy to mention the *MYCN* amplification typically confers a more aggressive course with evidence of early metastases, poor treatment response, and poor prognosis [16, 17]. SP-EPNs are, by definition, localized to the spinal cord, most frequently in the cervical spine, and lack the histologic features of MPE or SE [16, 18]. The median age at the time of diagnosis is approximately 41 years old, with a range between 11 and 59 years, and they typically confer a relatively benign course [16, 19]. Majority of SP-EPNs demonstrate loss of chromosome 22q where the *NF2* gene is located; however, it is not clear if there are other significant differences between *NF2*-altered SP-EPNs versus *NF2* wild-type SP-EPNs [16]. Additionally, only 31% of children with ependymomas show evidence of monosomy 22 [3].

Evidence supports the claim that pediatric ependymomas display a different genomic profile compared with that of adults, and tumor location also seems to be associated with differences in molecular genotypes. Given the lack of data on pediatric populations, much of the evidence we have to date is based on data from mixed age groups. Across all age groups, SP-EPNs harbor a gain of chromosome 7 in more than 95% of lesions and show loss of chromosome 6 and 22 in some tumors [3, 20]. Pediatric EPNs are more frequently associated with gain of chromosomes 1q, 7, and 9 and loss of chromosomes 1p, 3, 6/6q, 9p, 13q, 17, and 22 [3, 21]. Moreover, pediatric EPNs show a much higher association with gain of chromosome 1q compared with adults (20% versus 8%, respectively), as well as more partial and complex imbalances [21]. Of note, gain of chromosome 1q seems to be associated with more aggressive tumors and worse prognosis in intracranial ependymomas [3, 21, 22]. When comparing tumor location, EPNs arising in the spinal cord often demonstrate whole chromosomal imbalances including gain of chromosomes 7, 9, 11, 18, and 20 or loss of chromosomes 1, 2, and 10 versus intracranial ependymomas, which frequently harbor gain of chromosome 1q and loss of chromosomes 3, 9p, 13q, and 22 [21]. Furthermore, in contrast to intracranial ependymomas, SP-EPNS upregulate peptide production genes including *PLA2GS*, *ITIH2*, anteroposterior development homeobox (HOX) genes, and tumor suppressor gene *CDKN2A* [21].

In our case, cytogenetic and molecular studies of the tumor revealed an abnormal female karyotype with gain of chromosome 7, loss of chromosome 22, and chromothripsis of the entire chromosome 6. Chromothripsis is thought to be the result of a single event by which up to hundreds of clustered chromosomal rearrangements occur in localized, genomic regions of one or a few chromosomes. Such an event has the potential to hasten oncogenesis via creation of fusion genes, elimination of tumor suppressor genes, and loss of regulatory genes, which are critical for cellular proliferation and survival. This event was initially estimated to occur in approximately 2–3% of all cancers with a predilection for bone cancers; however, more recent studies suggest that the prevalence may be much higher with this phenomenon occurring across many cancer subtypes [23–26]. The literature regarding the prevalence of chromothripsis among pediatric tumors is limited. However, Grobner *et al.* [26] analyzed the molecular footprint of 961 tumors, which included 24 types of cancer and reported that 3 out of 32 mutational signatures were linked to chromothripsis and *TP53* mutations. Another study by Voronina *et al.* [25] reported similar findings that germline mutations in *TP53* and *ATM* are strongly linked with chromothripsis. Our patient declined sequencing analysis of the tumor; therefore, it remains uncertain if these genes harbor any somatic mutations.

There are rare reports of chromothripsis occurring in intracranial ependymomas. Zschernack *et al.* [27] reported chromothripsis of chromosome 22 in two astroblastoma-like tumors out of 18 pediatric non-v-rel avian reticuloendotheliosis viral oncogene homolog A (RELA)/non-yes-associated protein (YAP) supratentorial ependymomas. However, there are no case reports of chromothripsis occurring in SP-EPNs. We believe this case report is the first of its kind and, thus, a valuable addition to the molecular genetic signature of pediatric SP-EPNs. It is noteworthy to mention that both of our patient’s tumors, meningioma, and spinal ependymoma harbored genomic alterations that are commonly seen in these lesions except for chromothripsis of chromosome 6 in the SP-EPN.

Given the relative paucity of these tumors, especially in children, the true incidence and molecular characteristics are difficult to completely ascertain. More research is needed to further classify these tumors in the pediatric population as well as the population of patients with NF2 to better understand the nature of these entities as well as provide timely and effective treatment options.

This case also highlights the versatility and importance of single-nucleotide polymorphism microarray analysis on a FFPE tumor when a fresh sample is limited or not available. We believe the information acquired from the utilization of this tool is invaluable, and characterization of the tumor genomic profile may aid in future risk stratification and treatment.

## Data Availability

Not applicable.

## Abbreviations

AP: Anterior–posterior
ATM: Ataxia telangiectasia mutated
CC: Cranial–caudal
CDKN2A: Cyclin dependent kinase inhibitor 2A
CNS: Central nervous system
EPNS: Ependymomas
FFPE: Formalin-fixed paraffin-embedded
FISH: Fluorescence in situ hybridization
HIT: Hirntumor
HOX: Homeobox
ITIH2: Inter-alpha-trypsin inhibitor heavy chain H2
MEK: Mitogen-activated protein kinase enzyme
MPE: Myxopapillary ependymoma
MRI: Magnetic resonance imaging
mTOR: Mammalian target of rapamycin
MYCN: N-myc protooncogene protein (basic helix–loop–helix protein 37)
NF2: Neurofibromatosis type 2
PLA2GS: Phospholipase A2 group IIA
RELA: V-rel avian reticuloendotheliosis viral oncogene homolog A
SE: Subependymoma
SEER: Surveillance, Epidemiology, and End Results
SP-EPN: Spinal ependymoma
SP-MYCN: Spinal ependymoma with *MYCN* amplification
TP53: Tumor protein 53
TRV: Transverse
WHO: World Health Organization
YAP: Yes-associated protein

## References

[1] Taylor MD, Poppleton H, Fuller C (2005). Radial glia cells are candidate stem cells of ependymoma [published correction appears in Cancer Cell. 2006 Jan;9(1):70]. Cancer Cell.

[2] Cacciotti C, Fleming A, Ramaswamy V (2020). Advances in the molecular classification of pediatric brain tumors: a guide to the galaxy. J Pathol.

[3] Yang I, Nagasawa DT, Kim W (2012). Chromosomal anomalies and prognostic markers for intracranial and spinal ependymomas. J Clin Neurosci.

[4] Khalid SI, Kelly R, Adogwa O (2018). Pediatric spinal ependymomas: an epidemiologic study. World Neurosurg.

[5] Ellison DW. Central Nervous System Tumours: WHO Classification of Tumours, 5th ed. Lyon, France: World Health Organization; 2021. https://tumourclassification.iarc.who.int/chaptercontent/45/247. Accessed 23 Jan 2023.

[6] Hübner JM, Kool M, Pfister SM, Pajtler KW (2018). Epidemiology, molecular classification and WHO grading of ependymoma. J Neurosurg Sci.

[7] Chandler ME, Yunis JJ (1978). A high resolution in situ hybridization technique for the direct visualization of labeled G-banded early metaphase and prophase chromosomes. Cytogenet Cell Genet.

[8] Asthagiri AR, Parry DM, Butman JA (2009). Neurofibromatosis type 2. Lancet.

[9] Evans DG (2009). Neurofibromatosis type 2 (NF2): a clinical and molecular review. Orphanet J Rare Dis.

[10] Ardern-Holmes S, Fisher G, North K (2017). Neurofibromatosis type 2. J Child Neurol.

[11] Coy S, Rashid R, Stemmer-Rachamimov A, Santagata S (2020). An update on the CNS manifestations of neurofibromatosis type 2 [published correction appears in Acta Neuropathol. 2019 Aug 20]. Acta Neuropathol.

[12] Tamura R (2021). Current understanding of neurofibromatosis type 1, 2, and schwannomatosis. Int J Mol Sci.

[13] Hsu W, Jallo GI (2013). Pediatric spinal tumors. Handb Clin Neurol.

[14] Campian J, Gutmann DH (2017). CNS tumors in neurofibromatosis. J Clin Oncol.

[15] Benesch M, Weber-Mzell D, Gerber NU (2010). Ependymoma of the spinal cord in children and adolescents: a retrospective series from the HIT database. J Neurosurg Pediatr.

[16] Kresbach C, Neyazi S, Schüller U (2022). Updates in the classification of ependymal neoplasms: the 2021 WHO Classification and beyond. Brain Pathol.

[17] Ghasemi DR, Sill M, Okonechnikov K (2019). MYCN amplification drives an aggressive form of spinal ependymoma. Acta Neuropathol.

[18] Plotkin SR, O'Donnell CC, Curry WT, Bove CM, MacCollin M, Nunes FP (2011). Spinal ependymomas in neurofibromatosis type 2: a retrospective analysis of 55 patients. J Neurosurg Spine.

[19] Pajtler KW, Witt H, Sill M (2015). Molecular classification of ependymal tumors across all CNS compartments, histopathological grades, and age groups. Cancer Cell.

[20] Hirose Y, Aldape K, Bollen A (2001). Chromosomal abnormalities subdivide ependymal tumors into clinically relevant groups. Am J Pathol.

[21] Kilday JP, Rahman R, Dyer S (2009). Pediatric ependymoma: biological perspectives. Mol Cancer Res.

[22] Korshunov A, Witt H, Hielscher T (2010). Molecular staging of intracranial ependymoma in children and adults. J Clin Oncol.

[23] Stephens PJ, Greenman CD, Fu B (2011). Massive genomic rearrangement acquired in a single catastrophic event during cancer development. Cell.

[24] Cortés-Ciriano I, Lee JJ, Xi R (2020). Comprehensive analysis of chromothripsis in 2,658 human cancers using whole-genome sequencing [published correction appears in Nat Genet. 2020 May 13]. Nat Genet.

[25] Voronina N, Wong JKL, Hübschmann D (2020). The landscape of chromothripsis across adult cancer types. Nat Commun.

[26] Gröbner SN, Worst BC, Weischenfeldt J (2018). The landscape of genomic alterations across childhood cancers. Nature.

[27] Zschernack V, Jünger ST, Mynarek M (2021). Supratentorial ependymoma in childhood: more than just RELA or YAP. Acta Neuropathol.

